# The Opsonic Theory and the Resulting Method of Treatment of Bacterial Diseases

**Published:** 1907-03

**Authors:** 


### The Opsonic Theory and the Resulting Method of Treatment of Bacterial Diseases.

---

We have set aside all other material for special editorial expression this month in order to concentrate the mind of the reader upon this, one of the great discoveries in medical science in modern times. This subject has been a prominent one in medical periodicals for the past four months, but it has always been stated with so much of the technical verbiage of the laboratory that many who have attempted to read it have obtained but a dim idea of it. It is our desire to state the subject as clearly as possible in well-known terms, so that the physician may understand it whether he completed his medical college course last May or forty years ago.

When Pasteur began to realize the broad domain of the subject of the causation of disease by micro-organisms, and saw the effect of the use of a certain vaccine upon one of them (smallpox), he ventured the prediction that in time we would treat all bacterial diseases with their appropriate vaccines. This has been the goal which bacteriologists, pathologists and experimental therapeutists have had in view for many years, apparently getting nearer to it from year to year as errors in theory and method have been detected and corrected. One of the most successful of our experimenters has been Sir Almroth E. Wright of London, who, aided by Drs. G. W. Ross, Stewart R. Douglas, J. Freeman and other enthusiastic assistants, has been working heroically for several years. The result has been published over Dr. Wright's name in various papers and lectures under the general title "The Opsonic Index."

It has been noted for many years that when bacteria invade the tissues the system at once endeavors to combat them by means of the white blood corpuscles, which envelop the invading bacteria and destroy them. (This is the well-known process called phagocytosis—the destruction of the germs by the phagocytes.) However, in very many cases the invading army of bacteria is victorious and prevails over the defensive action of the white blood corpuscles.

The reason why either the bacteria, on the one hand, or the white blood corpuscles, on the other, are not always victorious has been the subject of much theorizing. Dr. Wright has discovered that there is in the blood serum or plasma of each individual a substance which prepares the bacteria for their destruction by the leucocytes. This he has termed an opsonin from the Latin word *opsono,* meanin "I prepare food." There is a different opsonin in the same blood affecting different bacteria. The blood of one individual will be stronger or weaker in opsonic power, or the blood of the same individual will be at different times stronger or weaker in opsonic power, in regard to each disease germ. The degree of opsonic power, ascertained by an examination of the blood, according to Dr. Wright's experiments, constitutes the patient's opsonic index for that disease.

The practical application of this theory is about as follows: A patient about to undergo treatment for an infection by a certain specific disease germ is tested for his opsonic index by a suitable examination of a specimen of his blood. According to the result of this examination his index of resistance is determined as being a certain per cent above or below normal. According to such determination, he is given a hypodermic injection (vaccination) from a previously prepared culture of that disease germ. He returns two or three times a week for a new test of his blood and a new vaccination according to what is shown by the test, the object being to keep the blood serum in a condition to prepare the bacteria for destruction by the white blood cells as rapidly as possible—that is, in as high a state of opsonic power as possible.

This theory has been put into very successful practical application in staphylococcic infection, by which acne, boils, sycosis and other cases of obstinate staphylococcic infection have been rapidly cured; in cases of lupus, tuberculosis of the joints, tuberculous glands and other forms of tuberculosis, with very satisfactory results; in empyema from pneumococcic infection, with rapid cure; in ulcerative endocarditis from streptococcus infection, with complete recovery, and in several other forms of specific bacterial infection.

A curious phase of the subject is that the same person may have a high opsonic power to some disease germs and a very low one against others at the same time. The apparent degree of health seems to give no indication as to the resisting power of the individual to a given disease germ. The opsonic index alone will give the proper indication.

We omit the details of the laboratory processes, as that would be entirely unsuitable in a presentation of this kind. The practician who wishes to enter into the subject practically will be compelled to familiarize himself with bacteriologic work. While this method of treatment is out of the reach of the average practicing physician at the present time, it is not too much to say that this principle will hereafter dominate the entire progress in the therapeutics of bacterial diseases, and that the physician who wishes to be in the advance ranks of the profession must prepare himself for this method of diagnosis and treatment. However, a great deal will yet be done by the special laboratory workers to bring the means of treatment according to the opsonic theory more nearly within the reach of the more intelligent practicing physician.

As a result of this new development, it would seem that the surgeon, who has had, since the discovery of antisepsis and aseptic procedure, a great apparent advantage over the physician, must now look well to his laurels, since many of the conditions for which surgical interference has seemed to be the only remedy are now amenable to this, which may be defined as merely a method of helping the patient in his efforts to overcome the disease.

Natural science was revolutionized by the Darwinian theory of natural selection and the survival of the fittest. It is possible that the science of the bacterial diseases has now reached its point of practical solution.—*Editorial in Medical Council, Philadelphia.*

